# Role of particle shape anisotropy on crack formation in drying of colloidal suspension

**DOI:** 10.1038/srep30708

**Published:** 2016-08-01

**Authors:** Venkateshwar Rao Dugyala, Hisay Lama, Dillip K. Satapathy, Madivala G. Basavaraj

**Affiliations:** 1 Polymer Engineering and colloid Science Lab, Department of Chemical Engineering, Indian Institute of Technology Madras, Chennai, 600036, India; 2 Soft Materials Laboratory, Department of Physics, Indian Institute of Technology Madras, Chennai, 600036, India

## Abstract

Cracks in a colloidal film formed by evaporation induced drying can be controlled by changing drying conditions. We show, for the first time that the crack morphologies in colloidal films are dependent on shape of constituting particles apart from the microstructure and particle assembly. In order to investigate the particle shape effect on crack patterns, monodispered spherical and ellipsoidal particles are used in sessile drop experiments. On observing the dried sessile drop we found cracks along the radial direction for spherical particle dispersions and circular crack patterns for ellipsoidal particle dispersions. The change in crack pattern is a result of self assembly of shape anisotropic particles and their ordering. The ordering of particles dictate the crack direction and the cracks follow the path of least resistance to release the excess stress stored in the particle film. Ellipsoids having different aspect ratio (~3 to 7) are used and circular crack patterns are repeatedly observed in *all* experiments.

The morphology and properties of particulate films formed upon evaporation of colloidal suspensions have various important technological applications in optical, electrical and medical fields^1^,^2^,^3^,^4^. Interestingly these dried colloidal films very often accompany spatially periodic cracks which can be of different morphologies^1^. Different evaporation techniques such as sessile drop evaporation^5^,^6^,^7^, drying under confinement^8^,^9^, vertical deposition^10^,^11^,^12^,^13^, etc, have been used to tailor and achieve different types of particulate films. Moreover, different model systems have been studied to understand the crack nucleation and their propagation during drying^8^,^14^,^15^,^16^. Allian and Limat^8^ studied the crack patterns by preparing colloidal films using directional drying of the suspension (confined between two glass plates) and found that crack spacing varies non linearly with the film height^8^. Sessile drop evaporation of suspension of spherical particles dried under isotropic drying conditions, mostly form particulate films with radial cracks. During the evaporation of sessile drop, the dispersed particles accumulate at the drop edge due to outward radial flow^17^. During this process, the stress develops in both radial (in the drying front direction) and perpendicular to radial direction (tangential direction to the sessile drop circumference). Finally, when the accumulated stress exceeds the critical value (*σ*_*c*_) along a particular direction, the cracks appear perpendicular to that direction. It is commonly accepted that drying of a colloidal suspension leads to an increase in stress within the film and the moment it exceeds *σ*_*c*_, the excess stress is released by virtue of crack formation. In isotropic evaporation, the dependence of crack morphologies on salinity of suspension^14^, size of the particle^15^, relative humidity^16^ and effect of substrate^18^ is well studied. Several analytical^8^,^19^ and computational models^20^,^21^,^22^ have been proposed to explain different crack morphologies as a function of film height and ordering of spherical particles in the film. However it is shown that these cracks can be suppressed by adding polymers thereby inducing depletion interaction^23^. But, the dependence of particle shape on the crack morphology is not addressed yet. The growing importance of particulate films consisting of shape anisotropic particles which often accompany cracks^9^,^11^ motivates us to investigate the correlation between particle shape and crack morphology.

In the present work, we study the morphology of the particle deposits and the resulting crack patterns by considering evaporation of sessile drops consisting of charged spherical (silica) and ellipsoidal (hematite) particles. Upon the evaporation of sessile drop on a solid substrate, ring like patterns similar to coffee ring is observed^17^. In such coffee-ring like particulate deposits, cracks are invariably formed. In case of suspension of spherical particles, cracks along the radial direction are observed in line with reported results and the crack morphology is attributed to the packing and ordering of particles^24^. For dispersions containing ellipsoid particles, we observed cracks in the form of concentric circles. In this report, we show the dependence of crack morphology on particle shape anisotropy apart from the particle ordering and their arrangements^25^. The direction of crack propagation is dictated not only by the direction of propagation of drying fronts but also by the particle self-assembly and the cracks propagate through a least resistance path^26^. The effects of particle concentrations and aspect ratios on crack morphologies are also studied.

## Results

### Crack formation with spherical and ellipsoidal particles

**Figure 1:**
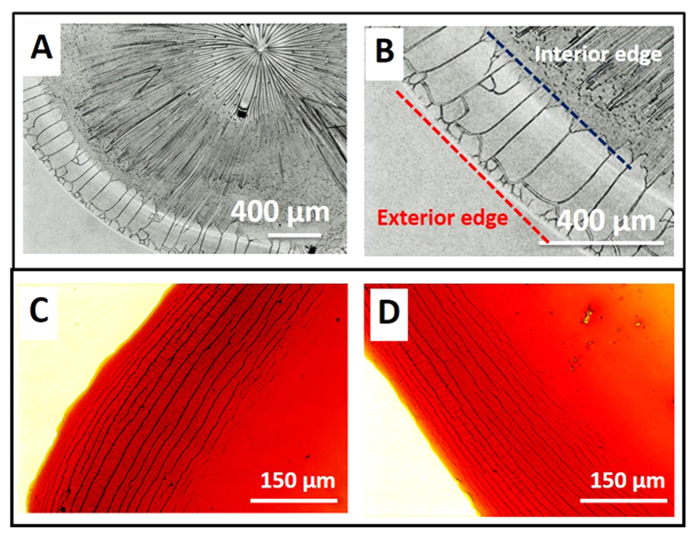
(**A,B**) Optical microscopy images of dried ring region consisting of colloidal silica particle of spherical shape with a diameter of 32 nm (Ludox TM-50). Cracks are oriented in radial direction. The crack spacing is estimated to be ~136 ± 31.61 *μm*. (**C,D**) Optical microscopy images of dried ring region of different locations consisting of hematite ellipsoids of aspect ratio 3.73 ± 0.37. Circular cracks are observed along the ring periphery. Crack spacing is estimated to be ~31.21 ± 7.72 *μm*.

Upon complete evaporation of sessile drop containing spherical particles (Ludox TM-50), a coffee-ring like pattern with cracks along radial direction are observed. These crack patterns are shown in Fig. 1A. From Fig. 1A, the cracks are found to be oriented in the radial direction with crack spacing of ~136 ± 31.61 *μm*. The observed patterns are very similar to that reported in past for spherical particle dispersions^14^,^16^. A closer look at the ring region reveals that the crack morphology have both ordered cracks at interior edge of the ring and disordered cracks at exterior edge as shown in Fig. 1B. The change in the crack morphology along the ring width is attributed to the uneven film height and particle ordering.

In order to understand the shape effect on crack patterns, a similar type of sessile drop evaporation experiment with dispersion of hematite ellipsoids (3.0 wt% and with an aspect ratio of 3.73 ± 0.37) is conducted under the favorable conditions where they form coffee-ring like deposits^7^,^27^. The coffee-ring like formation with hematite ellipsoids has been studied in more detail by Dugyala *et al*.^7^. It is shown that the particle-particle, particle-substrate and image charge interactions play a major role in the formation as well as the suppression of coffee-ring effect. The surface morphology of these deposits and the corresponding optical microscopy images are shown in Fig. 1C,D. In stark contrast to the previous spherical particle case, we found “cracks in the form of concentric circles” rather than cracks along the radial direction. These cracks are parallel to each other with an average crack spacing of ~31.21 ± 7.72 *μm*. The cracks in the middle region of the ring are continuous whereas near the exterior and interior edge are discontinuous.

### Effect of particle aspect ratio and concentration

**Figure 2:**
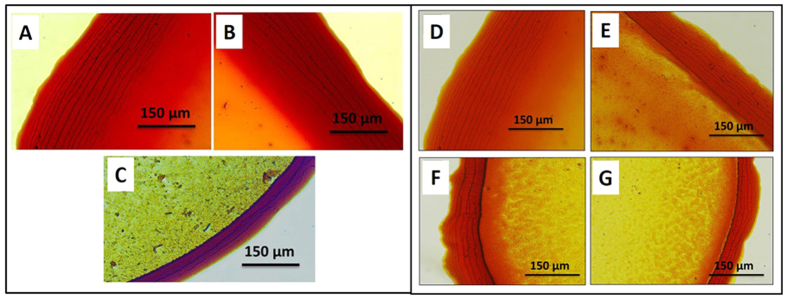
(**A–C**) Optical microscopy images of circular crack patterns observed with different aspect ratio particle - (**A**) aspect ratio 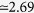 (4.34 wt% particle concentration); (**B**) aspect ratio 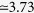 (3 wt% particle concentration); (**C**) aspect ratio 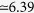 (1 wt% particle concentration). (**D–G**) Optical microscopy images of ring region for particles having aspect ratio 3.73 ± 0.37 with (**D**) 3 wt% (**E**) 2.25 wt% (**F**) 1.5 wt% (**G**) 0.75 wt% particle concentrations. For all particle concentrations reported here (from 0.75–3 wt%), circular cracks are observed. The ring width and crack spacing decrease as the particle initial concentration is decreased and the crack density (number of cracks per unit length) decreases as the particle concentration is increased.

We studied the crack formation by performing sessile drop evaporation experiments with colloidal dispersions containing hematite ellipsoids of three different aspect ratios (2.69, 3.73 and 6.34) and four different concentrations (0.75–3 wt%). In total, about sixty sessile drop evaporation experiments are performed. The optical microscopy images of crack patterns at different aspect ratio are shown in Fig. 2. Cracks in the form of concentric circles are always observed for the hematite ellipsoids of different aspect ratio that are studied. The particles are observed to be closely packed throughout the particulate film (SEM images are not shown here). On average, the major axis of the particle is found to be parallel to the crack direction. From the experiments, it is evident that the particle ordering significantly influence the crack patterns for anisotropic particles.

The optical microscopy images of crack patterns at different particle concentration are shown in Fig. 2. The corresponding crack spacing (distance between two cracks) and crack density (number of cracks per unit length) as a function of particle concentration are given in Table 1. It is observed that as the particle concentration increases the crack spacing also increases (i.e, crack density reduces) followed by the increase in height at the edge due to accumulation of more particles. In case of higher weight fraction suspension, fewer cracks with larger crack widths are formed to release the excess stress. On the other hand for low weight fraction suspension, a larger number of cracks with smaller width are formed. The variation of crack spacing as a function of initial particle concentration is shown in Table 1 and it is evident that the crack spacing is proportional to the initial particle concentration. We conjecture that this behavior is related to the large height of the ring formed at high particle concentration.

### Self assembly of particles at the vicinity of cracks

**Figure 3:**
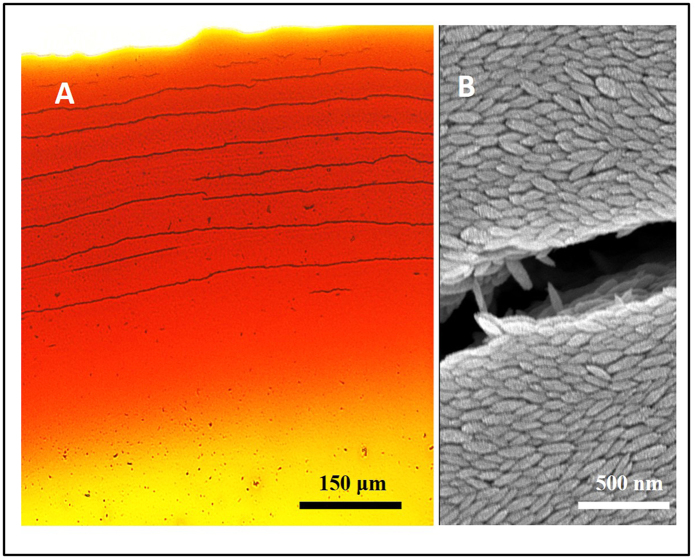
The optical microscopy image of the ring region (**A**) and the SEM image in the vicinity of cracks (**B**). The aspect ratio of the particles is 3.73 ± 0.37 and the particle concentration is 3.0 wt.%.

In order to understand and investigate the effect of self-assembly/particle ordering on crack patterns, we have recorded scanning electron micrographs of the area near cracks, as shown in Fig. 3B. From SEM micrograph it is clear that, ellipsoidal particles are arranged in a closed packed structure with major axis of the particle parallel to the crack direction. A similar ordered arrangement of ellipsoidal particles in particulate films fabricated via convective and vertical deposition have been reported^9^,^11^. We have recently shown that the ordering and hence the particle arrangement in the film is dictated by particle surface charge^11^. The particle self assembly near the edge depends on the ratio of particle diffusivity length scale to the hydrodynamic length scale^6^. If this ratio is lower than one, the radial velocity of fluid is sufficiently low and particles reaching the contact line have enough time to arrange into ordered structures. Otherwise, the radial flow velocity is sufficiently high and the particles do not have enough time to reassemble and this leads to disorder arrangement near the edge. Marin *et al*. observed an order to disorder transition with spherical particles within the ring and the ordering increased with decreasing particle size^6^. In case of nano size ellipsoidal particles, the hydrodynamic torque exerted on the one end of a particle due to the radial fluid flow turns the particles parallel to the contact line^27^. It is evident from the SEM image that particles major axes are parallel to each other and their alignment is parallel to the crack direction. The optical microscopy and SEM images for dried droplets of colloidal dispersions hematite ellipsoids with aspect ratio 2.69, 3.73 and 6.3 are included in the supporting information.

## Discussion

**Figure 4:**
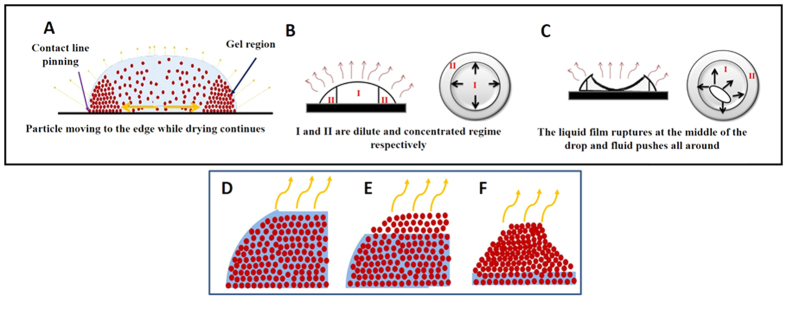
Schematic diagram of sessile drop evaporation at different stages. (**A**) Initial drying stage, particles are accumulated near the contact line due to capillary flow. (**B**) Side and top view of sessile drop, where I and II are dilute and concentrated (gel) regions, respectively. (**C**) Rupture stage- rupturing of liquid film inside the droplet. The arrow indicates the outward flow direction of the remaining water in the film. (**D–F**) Schematic depicts shrinking of colloidal particle during final stages of drying. Final drying stage - water in the ring region starts to evaporate. Water evaporates from the top to the bottom along the ring height. During this stage the capillary pressure and van der Waals interaction hold the particles together.

The crack formation mechanism during the evaporation of sessile drop consisting of dispersion of spherical particles is fairly well established but for ellipsoid particles it is still not explored. It is known that in sessile drop evaporation, the solvent continues to evaporate from the drop surface and the evaporation rate is higher near to the three phase contact line because of the curvature of the drop. The nonuniform evaporation creates an outward radial flow during evaporation and the flow drags the particles to the drop edge^17^. The various stages involved in drying of a sessile droplet are shown schematically in Fig. 4(A–C). The initial drying stage comprises of pinning of the sessile drop followed by the evaporation of the fluid. During this stage the contact angle decreases with time and the dispersed particles are deposited near the contact line. This regime, designated by ‘II’ in Fig. 4B, is commonly known as the concentrated region or the gel region. The evaporation of the solvent from the gel region is replaced by the fluid from the central region ‘I’ of the drop. During this stage, the migration of particles will continue until the contact angle of the sessile drop becomes negligibly low (≤1°). As the water continues to evaporate the thin fluid film in the interior region of the drop ruptures (as shown in the top view of Fig. 4C). Soon after, the residual fluid in the thin film is pushed outwards as shown by the arrows in Fig. 4C (see supporting video). The nucleation of cracks at the interior of the gel region occurs when the ruptured fluid film first intersects the gel region. As the solvent evaporates further, the cracks spread throughout the circumference of the ring. During the final stage of drying (Fig. 4(D–F)), the water evaporation from the concentrated ring region leads to an increase in immersion capillary pressure and brings the particles together and causes a shrinkage in the film. The water meniscus recedes downward and after complete evaporation of fluid the particles are held together via the van der Waals forces. The capillary pressure is given as^2^


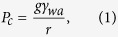


where, *γ*_*wa*_ is the water-air interfacial tension and *r* is the capillary pore radius and *g* is the geometric factor that depends on the meniscus geometry. Since the ellipsoid particle pack much closely than spherical one^28^, radius of the meniscus ‘*r*’ for ellipsoid particle assembly is smaller than their for the spherical counterpart. Thus the capillary pressure for the assembly of ellipsoid particles is higher.

**Figure 5:**
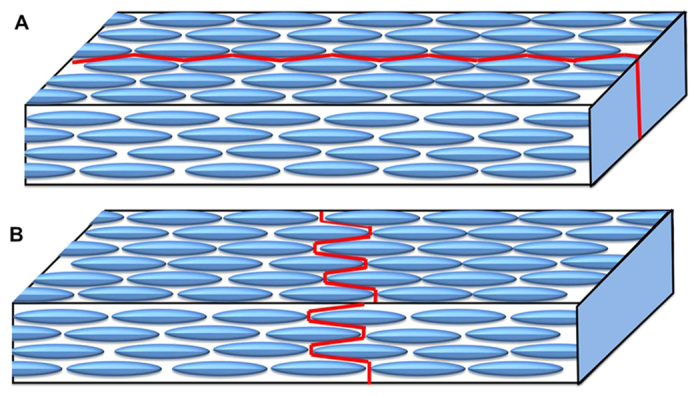
Schematic diagram of crack propagation in assembly of ellipsoids. (**A**) straight crack propagation (**B**) tortuous crack propagation.

During the final stage of drying, the tensile stress builds up within the film due to the compression of particle assembly as a result of capillary pressure. When the stress exceeds its critical value *σ*_*c*_, the excess stress is released via the nucleation of cracks at the film surface. It is known that, for brittle materials, crack propagation occurs by breaking of bonds between the atoms^29^. Similarly, for particulate films the crack propagation can be considered as detachment of colloidal particles or breaking of the contact between the particles. An ordered array of spherical particles having isotropic packing density will *not* have any preferred fracture direction. In contrast for an ordered assembly of anisotropic particles the crack propagation will have a preferred direction depending on the particle ordering. Our experimental observations unambiguously reveal the well ordered and closed pack arrangement of ellipsoids in the dried ring. Two possible scenarios that lead to circular cracks (Fig. 5A) and cracks along radial direction (Fig. 5B) in a dried drop of dispersions of ellipsoids are shown schematically. The crack path in Fig. 5A is rather straight whereas the path in Fig. 5B is tortuous. That is, the energy required to activate cracks along the straight path is certainly lower than the tortuous path. Hence straight cracks always form first to release excess stress in the ordered film of ellipsoidal particles. The crack direction observed in our experiments (SEM image shown in Fig. 3) is parallel to the major axis of ellipsoid which is similar to Fig. 5A. It is also evident from our experiments that the ordering of ellipsoids dictates the crack orientation. Similar experimental observations are reported for composite materials earlier^26^. In the past, several models are proposed where the cracks patterns are explained using bead-spring model in dense particulate films. Richardi *et al*. have shown through both simulation and experiments that ordering of particles leads to formation of ordered cracks^30^.

To summarize, we investigated the effect of particle shape on the crack morphology by using sessile drop evaporation of liquid drops containing spherical particles (Ludox TM-50) and ellipsoidal particles (hematite). We show that the crack patterns changed from *radial to circular* when the particle shape is changed from spheres to ellipsoids. Moreover, ellipsoidal particles are closely packed in the vicinity of cracks and the major axis of particles is parallel to the crack directions. In case of spherical particle film, the crack path does not have a preferred direction but however the crack direction is dictated by direction of drying front^31^. In case of ellipsoidal particle films, cracks are circular and are found to be dictated by particle shape and their arrangement. We have also studied the effect of aspect ratio of ellipsoids and their concentrations on the crack patterns. Irrespective of aspect ratio of ellipsoids that are studied, we observed circular crack patterns. The crack widths are found to depend on the initial concentration of particles. Our experimental results showing the crack patterns of shape anisotropic (hard) colloidal particle assembly may further enhance the understanding of the crack patterns in dried suspensions of biological fluids such as blood containing anisotropic soft colloidal particles^32^,^33^.

## Methods

### Synthesis and characterizations of hematite ellipsoids

Ellipsoidal hematite particles with controlled aspect ratio were synthesized by forced hydrolysis method as mentioned by Ocaña *et al*.^34^. Physical dimensions of hematite particles are tabulated in Table 2. One of the advantage of using the hematite particles is that their surface charge can be varied by changing pH of the suspension. Therefore, by adjusting the pH by the addition of either *HNO*_3_ or *NaOH*, the surface charge is varied and measured with electrophoretic dynamic light scattering technique (Nano partilce SZ-100, Horiba, Japan).

### Sessile drop evaporation method

In all the experiments, glass slides are used as a substrate and they are treated with pirahana solution (70% *H*_2_*SO*_4_ and 30% *H*_2_*O*_2_). A 2 *μl* stable dispersion of both hematite (aspect ratio 2.69, 3.73 and 6.30 and particle concentrations varying from 0.75 wt% up to 3.0 wt%) and Ludox TM-50 silica (of diameter 32 nm diluted to a particle concentration of 3.0 wt%) are placed on the cleaned glass slides and are allowed to evaporate at room temperature of 25 ± 2 °C with relative humidity of 65 ± 5%. Sessile drop evaporation experiments were conducted by using aqueous hematite suspensions that were initially at pH of 2.0. Under these conditions, the particles are highly charged with a zeta potential of −40 mV (in the presence of 0.01 M NaCl). Due to the small particle size and the high surface charge density, the settling time is much larger than the time scale of experiments^7^. The droplet takes approximately 15 minutes for complete drying. The sessile drop drying is monitored using the bright field inverted optical microscope (Leica, France) with 20X objective. After the complete evaporation of solvent, the microstructures are captured with microscopy. In order to visualize the arrangement of particles in the dried film at the length scale of particle size, a high resolution scanning electron microscope (SEM) (Hitachi, Japan) is used. The samples are sputter coated with gold for 30 seconds prior to SEM imaging.

## Additional Information

**How to cite this article**: Dugyala, V. R. *et al*. Role of particle shape anisotropy on crack formation in drying of colloidal suspension. *Sci. Rep*. **6**, 30708; doi: 10.1038/srep30708 (2016).

## Supplementary Material

Supplementary Information

Supplementary Movie S1

## Figures and Tables

**Table 1:** Crack spacing (*W*) and crack density as a function of particle weight fractions for particles having aspect ratio 3.73 ± 0.37.

S. No.	wt%	Crack spacing (*μm*) *W*	Cracks per unit length (*μm*^−1^)
1	3.00	31.21 ± 7.72	0.036
2	2.25	23.28 ± 0.95	0.055
3	1.50	22.10 ± 2.27	0.060
4	0.75	19.85 ± 0.75	0.076

The errors in crack spacing are estimated from the crack patterns recorded at multiple locations on the coffee-ring for three similar sessile drop experiments.

**Table 2:** Physical properties of hematite ellipsoids: dimensions and aspect ratio.

S. No.	Major axis, 2b (nm)	Minor axis, 2a (nm)	Aspect ratio, *α*
1	140.59 ± 10.72	52.84 ± 5.64	2.69 ± 0.39
2	192.37 ± 21.10	51.84 ± 5.55	3.73 ± 0.37
3	294.73 ± 20.62	46.71 ± 6.44	6.34 ± 0.77
