# Supplementary material for: Role of particle shape anisotropy on crack formation in drying of colloidal suspension
Source: Sci Rep. 2016 Aug 1;6:30708. doi: 10.1038/srep30708 (PMC4967893; doi:10.1038/srep30708)
Supplement: Supplementary Information [file srep30708-s1.pdf]

Supporting Information

Role of particle shape anisotropy on crack formation in drying of colloidal suspension

Venkateshwar Rao Dugyala[1+], Hisay Lama[2+], Dillip K Satapathy[2], Madivala G Basavaraj[1*]
PECS, Department of Chemical Engineering, Indian Institute of Technology Madras, Chennai, 600036, India
Department of Physics, Indian Institute of Technology Madras, Chennai, 600036, India
basa@iitm.ac.in

1. The optical microscope and SEM image of cracks

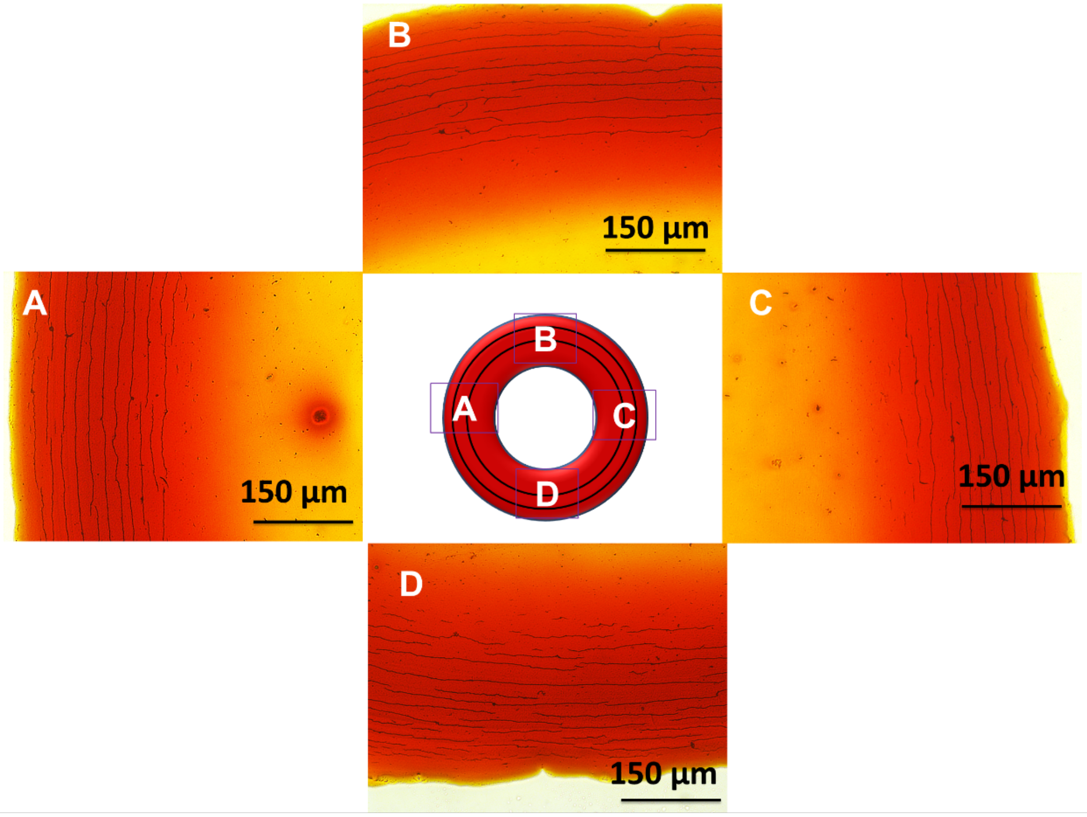

Figure S1: Optical microscope images of dried ring region at different locations containing hematite ellipsoids of aspect ratio ~ 2.7 (3.1 wt.% particle concentration).

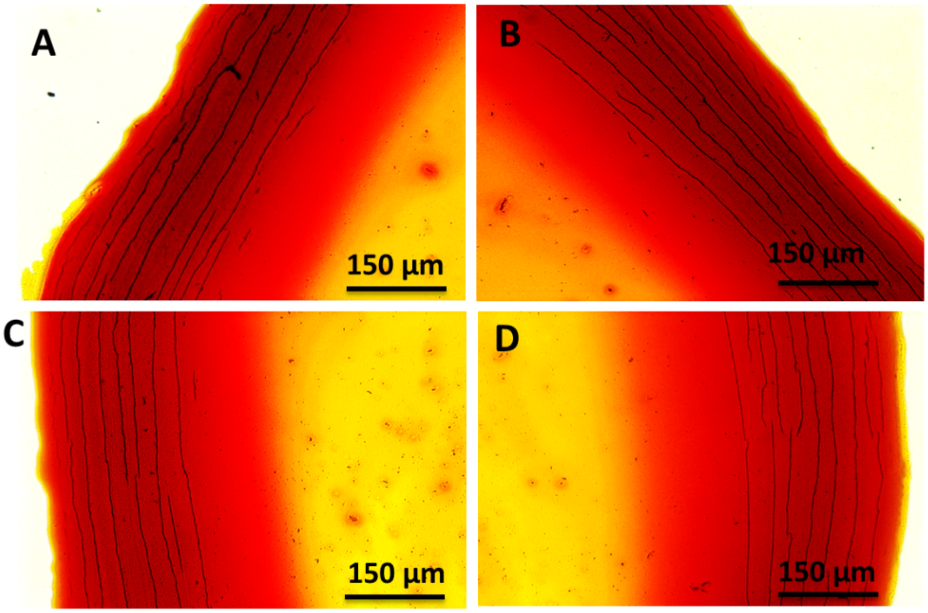

Figure S2: Optical microscope images of dried ring region at different locations containing hematite ellipsoids of aspect ratio ~ 2.7 (4.0 wt. % particle concentration).

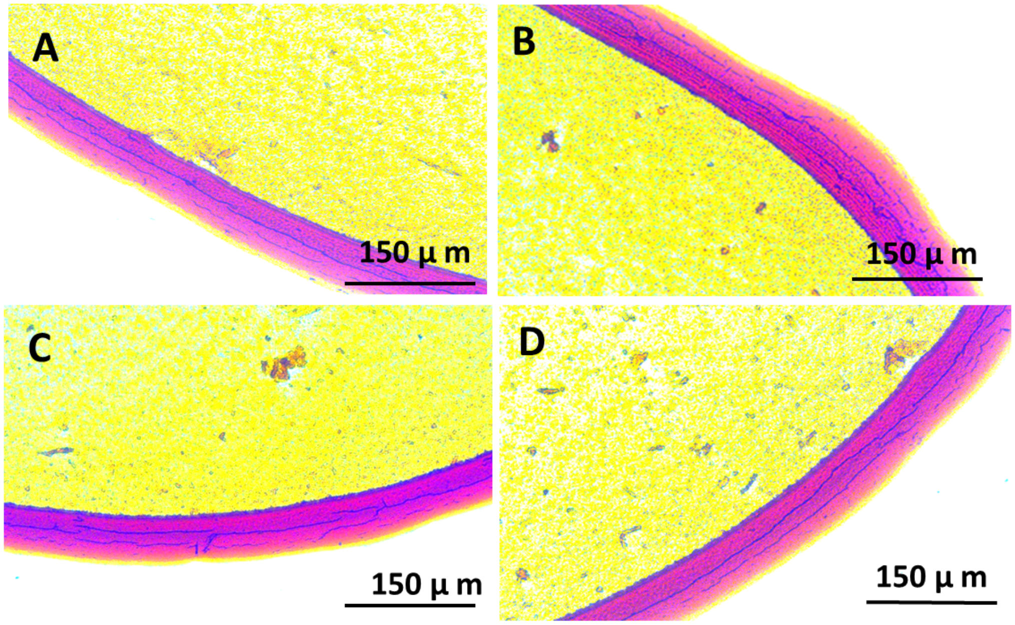

Figure S3: Optical microscope images of dried ring region at different locations containing hematite ellipsoids of aspect ratio ~ 6.4 (1 wt. % particle concentration).

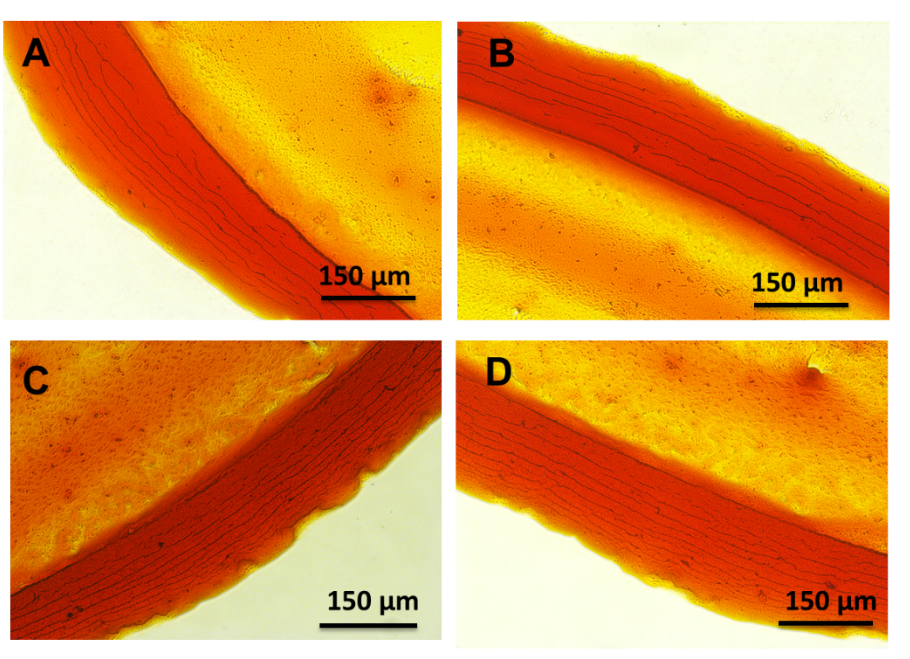

Figure S4: Optical microscope images of dried ring region at different locations containing hematite ellipsoids. (A-B) Aspect ratio ~ 2.7 (1.2 wt. % particle concentration) and (C-D) aspect ratio ~ 3.7 (2.3 wt. % particle concentration).

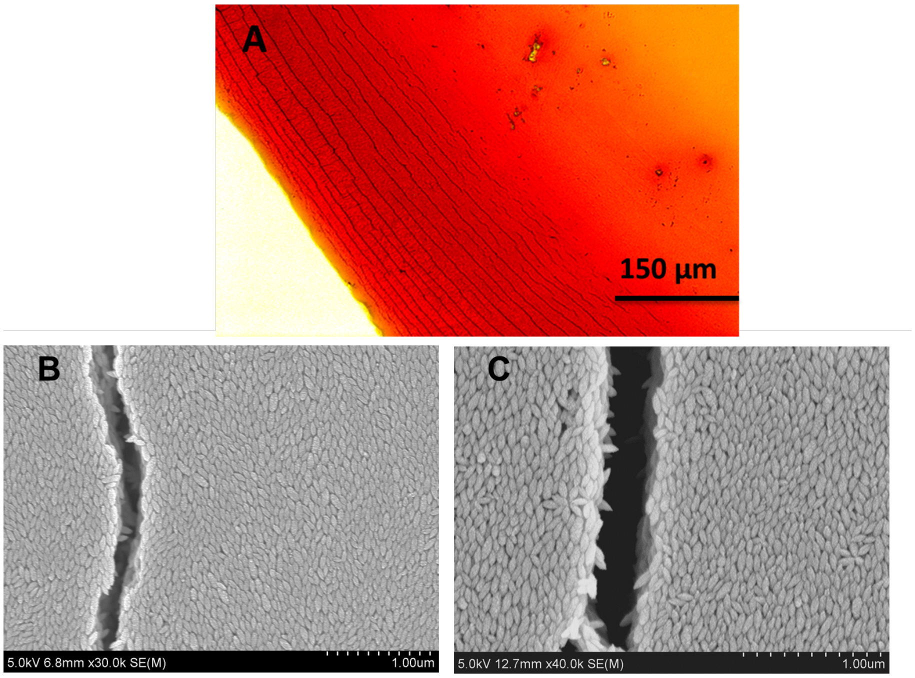

Figure S5. The optical microscope of the ring region (A) and SEM images at the vicinity of cracks (B-C). The aspect ratio of the hematite ellipsoids is ~ 2.7 (3.2 wt.% particle concentration).

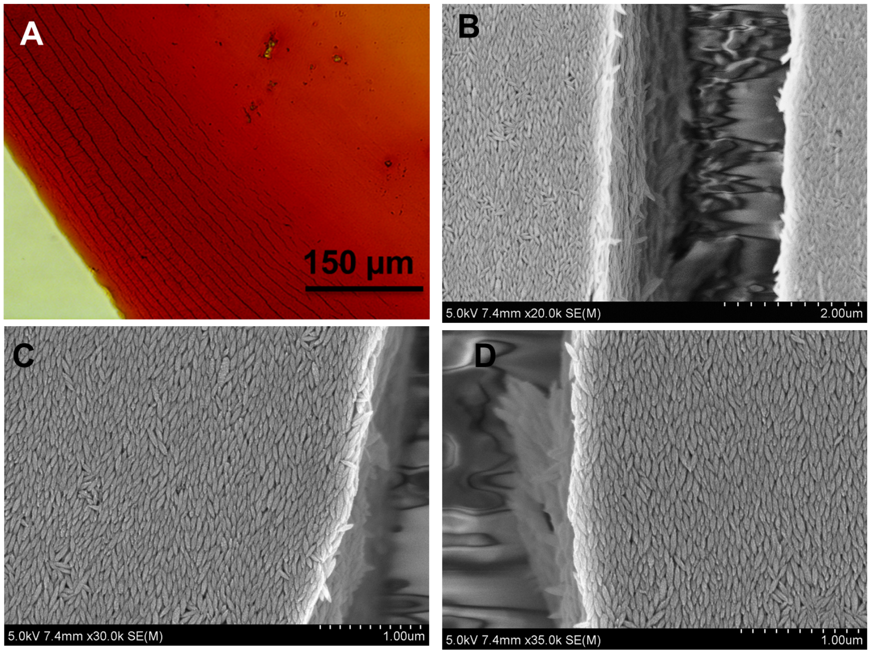

Figure S6. The optical microscope of the ring region (A) and SEM images at the vicinity of cracks (B-D). The aspect ratio of the hematite ellipsoids is ~ 3.7 (2.2 wt.% particle concentration).

2. Radial crack formation:

Movie. S1. The video shows the formation of cracks along the radial direction during the evaporation of sessile drop containing spherical particles. A crack is initiated when the thin fluid film in the interior region of the drop ruptures and thereafter, cracks propagate throughout the deposited ring as the liquid further evaporates. In these experiments, 2 μL aqueous drop (3 wt. %) of spherical colloidal silica of size 32 nm (Ludox TM-50) is dried on a cleaned glass slide.
